# Clinico-radiological Outcomes of Using Modified Stoppa Approach for Treating Acetabular Fractures: An Institutional Review

**DOI:** 10.7759/cureus.7821

**Published:** 2020-04-24

**Authors:** Mantu Jain, Pankaj Kumar, Sujit K Tripathy, Sudarsan Behera, Rajesh Rana, Sudhanshu Das

**Affiliations:** 1 Orthopaedics, All India Institute of Medical Sciences, Bhubaneswar, IND; 2 General Surgery, All India Institute of Medical Sciences, Bhubaneswar, IND; 3 Orthopaedics, All India Institute of Medical Sciences, Bhubaneshwar, IND; 4 Orthopaedics, Institute of Medical Sciences and SUM Hospital, Bhubaneswar, IND

**Keywords:** acetabulum, modified stoppa, matta, harris hip score

## Abstract

Introduction

Acetabular fractures are complex intra-articular fractures. The extra-pelvic ilioinguinal (IL) has been the workhorse for the anterior approach and remains the gold standard. The major difference between the IL and the Stoppa approaches is that Stoppa allows for the avoidance of the middle window of the IL approach. Hence, the modified Stoppa approach (MSA) can be adopted by a comparatively less experienced surgeon with minimal complications. The purpose of this study is to evaluate the radiological and functional outcomes of patients operated on using the MSA.

Materials and methods

Patients operated on by the MSA for acetabular fractures with a minimum of one year of clinical and radiographic follow-ups were reviewed. CT scans and radiographs were evaluated for the fracture pattern, time to surgery, operative time, blood loss, quality of reduction (Matta criterion), FO [Harris hip score (HHS) and Nach Merle d'Aubigné and Postel score (NMAPS)] and complications (perioperative and follow-up). Twenty-three of 26 patients with 45 acetabular fractures operated between January 2016 and November 2018 were included. Descriptive statistics were used for demographic data, and Pearson’s chi-squared statistic was calculated for the association between radiological and functional outcomes.

Results

Among the 23 patients, the mean age was 38.5 years (range: 15-65) with a male-to-female ratio of 18:5. The average time to surgery was 11.5 days (range: 2-32), operating time was 155 minutes (range: 90-243), and average blood loss was 650 ml (range: 500-1,250). A supplemental lateral window was used in 20 patients (87%), and three underwent the combined anterior and posterior [Kocher Langenbacks (KL)] approach. All cases were unilateral. The transverse fracture was the most common pattern (eight patients) followed by the associated both-column fracture in six and T-type, isolated anterior column fracture, and anterior column and posterior hemi-transverse fractures seen in three patients each. Iliac blade (high anterior column) fracture was seen in 14 cases and one patient had associated sacral type II fracture. Road traffic accidents accounted for 61% of the injuries and injury severity score (ISS) of >15 (polytrauma) was seen in more than 50% of the cases (associated with other organ injuries). The radiological outcome was anatomical in 52% of the cases, imperfect in 39%, and poor in 9%. The functional outcomes were good to excellent in 74% (HHS) and 79% (NMAPS) of the cases. The association and correlation between them were nonsignificant (p-value: >0.5). Two patients developed a superficial infection and three had iatrogenic obturator nerve palsy. One patient had a direct inguinal hernia, one had grade 3 bedsores, and two patients developed grade 2 arthritic changes during the follow-up. No case of vessel injury was encountered.

Conclusion

Adoption of the MSA for the treatment of acetabular fractures leads to a good-to-excellent anatomical reduction in most cases while providing direct visualization of the quadrilateral plate and posterior column. The learning curve is smaller for less-experienced surgeons in terms of complications and results. We recommend this technique as a viable alternative to the IL approach for anterior acetabular fixation.

## Introduction

Acetabular fractures are complex fractures that require much expertise to treat, especially surgically. Firm anatomical reduction, particularly of the weight-bearing dome, is the best option for a satisfactory functional outcome [1-2]. Judet et al. first classified these fractures and formulated the basic principles of management [3]. The approaches can be classified as anterior, posterior, and extensile even though combinations of these methods have been used by surgeons. The extra-pelvic ilioinguinal (IL) has been the workhorse of the anterior approach and remains the gold standard [4-5]. However, Hirvensalo et al. introduced the Stoppa intrapelvic approach in the early 1990s, which has gained a lot of popularity in recent years as it is an easier method and easily replicated by surgeons [6]. The significant difference between the IL and Stoppa approaches lies in bypassing the middle window and, thus, sparing the dissection of neurovascular structures (femoral nerve, external iliac vessels, and inguinal canal contents). Stoppa’s approach has been tailored and modified by various surgeons, and one of these modifications is the additional iliac window [7-9].

The purpose of this study was to audit the midterm results of operations performed using the modified Stoppa approach (MSA) by relatively less experienced surgeons as an anterior approach in the treatment of acetabular fractures and to compare the results with those of operations performed by more experienced and qualified surgeons.

## Materials and methods

Our study was an institutional, retrospective, and longitudinal one conducted at a premier tertiary level hospital from January 2016 to November 2018. Of the 45 patients with pelvic-acetabular fractures operated during the period, 26 patients had an anterior approach, of which three were excluded: two had undergone an IL approach and one had a follow-up of less than a year. Twenty-three patients met the inclusion criterion with at least a year of follow-up of clinical and radiographic results. Their data including the time interval between injury and surgery, approach (single or combined), operative time, blood loss, quality of reduction, functional outcomes, and perioperative complications were extracted from past medical records. The MSA advocated by Sagi et al. was used in all patients [9].

Approach

The patient was positioned supine on the operating table and the fracture reducibility was confirmed with fluoroscopy using traction. The lower abdomen, along with the involved lower limb, was prepared and draped to allow the hip to be flexed to relax the neurovascular structures, and a sandbag was also placed under the knee for the same purpose. The table was tilted ipsilaterally by 20-30 degrees and the surgeon placed himself on the contralateral side for "peeping inside". A 10-12-cm transverse incision was made about 2 cm proximal to the symphysis. The incision was deepened, and the rectus abdominis muscle was split midline. The ipsilateral rectus was then partly erased from the superior pubic ramus to ease the tension during retraction. The corona-mortis anastomosis was searched, identified, and ligated and, subsequently, subperiosteal dissection and release of the fascia were carried out to reach the fracture fragments. The obturator nerve and vessels were identified and protected. A lateral window was made along the iliac crest (third window of the IL approach) to expose the outer and lateral aspects and the anterior column fracture (exiting the iliac crest) or to fix the posterior column with a lag screw. Implants were inserted under direct vision and confirmed to be extra-articular by rotation head and fluoroscopy. Postoperatively, range-of-motion exercises were initiated using the continuous passive motion device except in patients with severe comminution of the quadrilateral plate where skeletal traction was applied for a further three weeks.

Assessment

Radiological assessments were made using the anteroposterior and Judet view in X-rays and CT images were studied for preoperative classification and postoperative outcomes. Fracture patterns were categorized as per the criteria laid down by Judet et al. [3]. Fracture reduction quality was assessed in three grades (anatomical: ≤1 mm, imperfect: 2-3 mm, and poor: ≥3 mm) of displacement as per the criteria given by Matta et al [2]. Functional outcomes were evaluated using the Harris hip score (HHS) and Nach Merle d'Aubigné and Postel score (NMAPS) [10-11].

Statistical analysis was done using R version 3.6.1. Demographic data were analyzed using descriptive statistics. Categorical variables were expressed as percentages, and numerical variables (non-parametric) were expressed as medians and interquartile The association and correlation between the radiological and functional outcomes were calculated using chi-square and Kendall correlations. A p-value of <0.05 was regarded as statistically significant.

## Results

The mean age of the patients at presentation was 38.5 years (range: 15-65) and there were 18 men and five women. The mean time interval between the injury and surgery was 11.5 days (range: 2-32). The average operating time was 155 minutes (range: 90-243), and the average blood loss was 650 mL (range: 500-1250). Road traffic accident was the most common mode of injury (n = 14, 61%), followed by fall from height (n = 7, 30%); worksite injury and sports injury occurred in one patient (4.5%) each. Patients also had associated injuries in the form of either head injury (n = 3), blunt trauma abdomen (n = 1), chest (n = 1), and extremity injury (n = 7), making more than half of them (n = 12) victims of polytrauma (ISS: ≥15).

All cases were unilateral. Associated both-column fracture was the most frequent type of fracture (n = 9, 40%), followed by transverse type fracture (n = 5, 22%), anterior column fracture (n = 4, 17%), anterior column and posterior hemi-transverse fracture (n = 3, 13%), and T type fracture (n = 2, 9%). There was an association of iliac wing (high anterior column) in many cases (n = 14), and one associated both-column fracture had associated sacral type II fracture.

A single approach (anterior MSA) was used in 20 patients, and a combined [anterior MSA and posterior Kocher Langenbeck (KL)] approach was followed in three patients. Patients with only anterior column fracture had their fracture fixed with at least one pelvic reconstruction plate, and patients with quadrilateral plate displacement required two plates (a combination of supra and infra pectineal plates or a recon plate/distal radius plate placed below a supra pectineal plate). Additional plates were used for the iliac wing if required. Three patients undergoing a combined approach were operated in the prone position (KL approach fixed with contoured plate) and flipped supine position for MSA. Two patients had the posterior column fixed with a single, long, partial threaded, cannulated cancellous screw. The outcome was evaluated using the Matta criteria for radiological and HHS and NMAPS for functional grading. The details of the patients are given in Table 1.

**Table 1:** Numerical data of patients showing radiological reduction and functional outcomes HHS: Harris hip score; NMAPS: Nach Merle d'Aubigné and Postel score

				Radiological reduction					
				Anatomical		Imperfect		Poor	
Functional outcome		HHS, n (%)	NMAPS, n (%)	HHS, n (%)	NMAPS, n (%)	HHS, n (%)	NMAPS, n (%)	HHS, n (%)	NMAPS, n (%)
Excellent	Acceptable	7 (31)	6 (26)	4 (17)	4 (17)	3 (13)	2 (9)	0	0
Good		10 (43)	12 (52)	5 (22)	7 (31)	4 (17)	4 (17)	1 (4)	1 (4)
Fair	Non-acceptable	4 (17)	3 (13)	3 (13)	1 (4)	1 (4)	2 (9)	0	0
Poor		2 (9)	2 (9)	0	0	1 (4)	1 (4)	1 (4)	1 (4)
Total		23 (100)		12 (52)		9 (39)		2 (9)	

**Figure 1:**
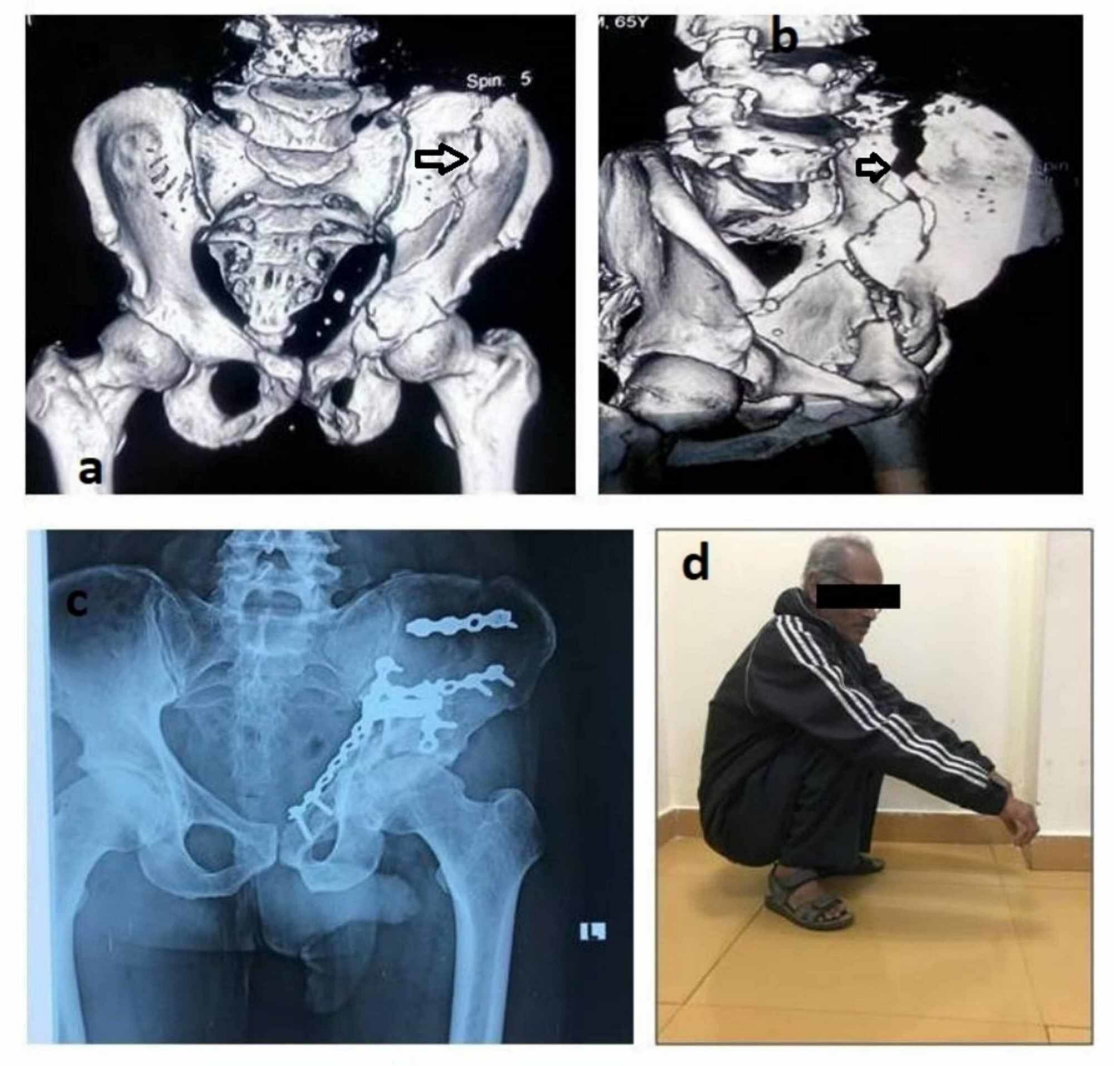
A case of anterior column fracture extending high in iliac blade (arrows) with final postoperative X-rays and functional outcome a, b: preoperative CT; c: postoperative radiograph; d: clinical photograph of the patient squatting CT: computed tomography

**Figure 2:**
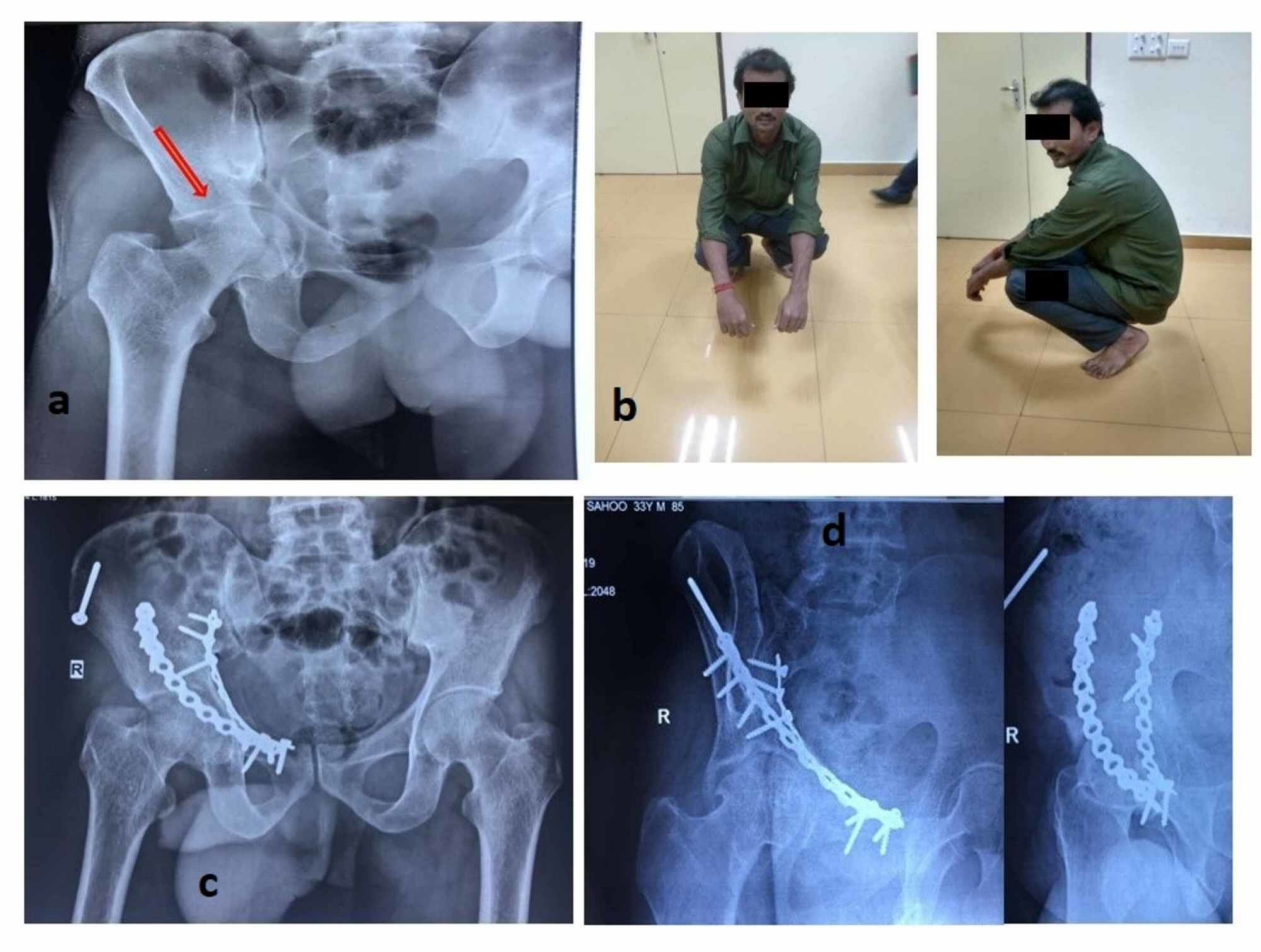
A case of transverse fracture showing head impaction (red arrow) with final postoperative X-rays and functional outcome a: preoperative radiograph; b: clinical functional outcome; c: follow-up radiograph anteroposterior view; d: follow-up radiograph iliac and obturator view

**Figure 3:**
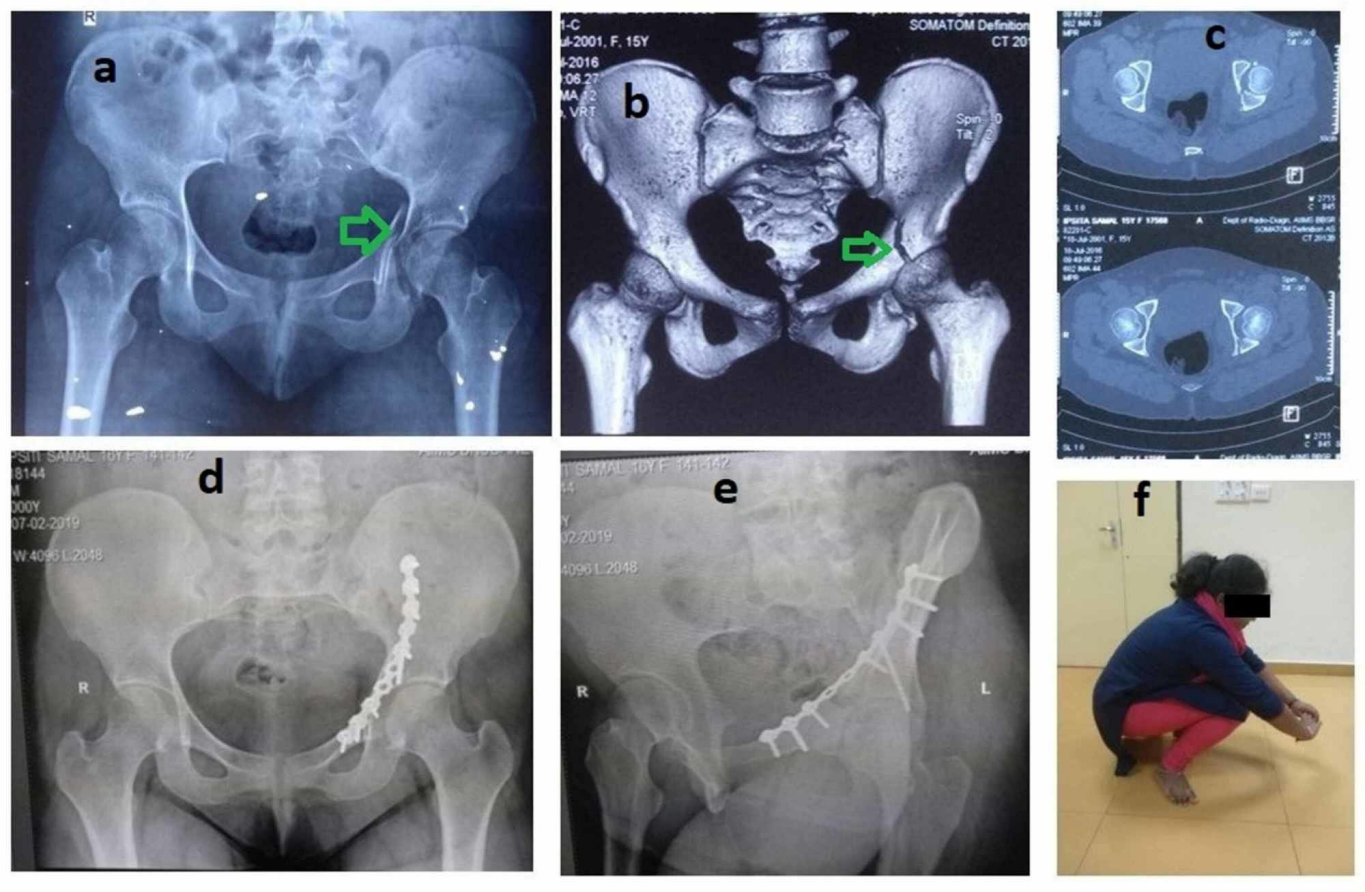
A case of transverse fracture (green arrows) with final postoperative X-rays and functional outcome a: preoperative radiograph; b, c: preoperative CT; d, e: two-year follow-up radiograph; f: clinical photograph of the patient squatting CT: computed tomography

**Figure 4:**
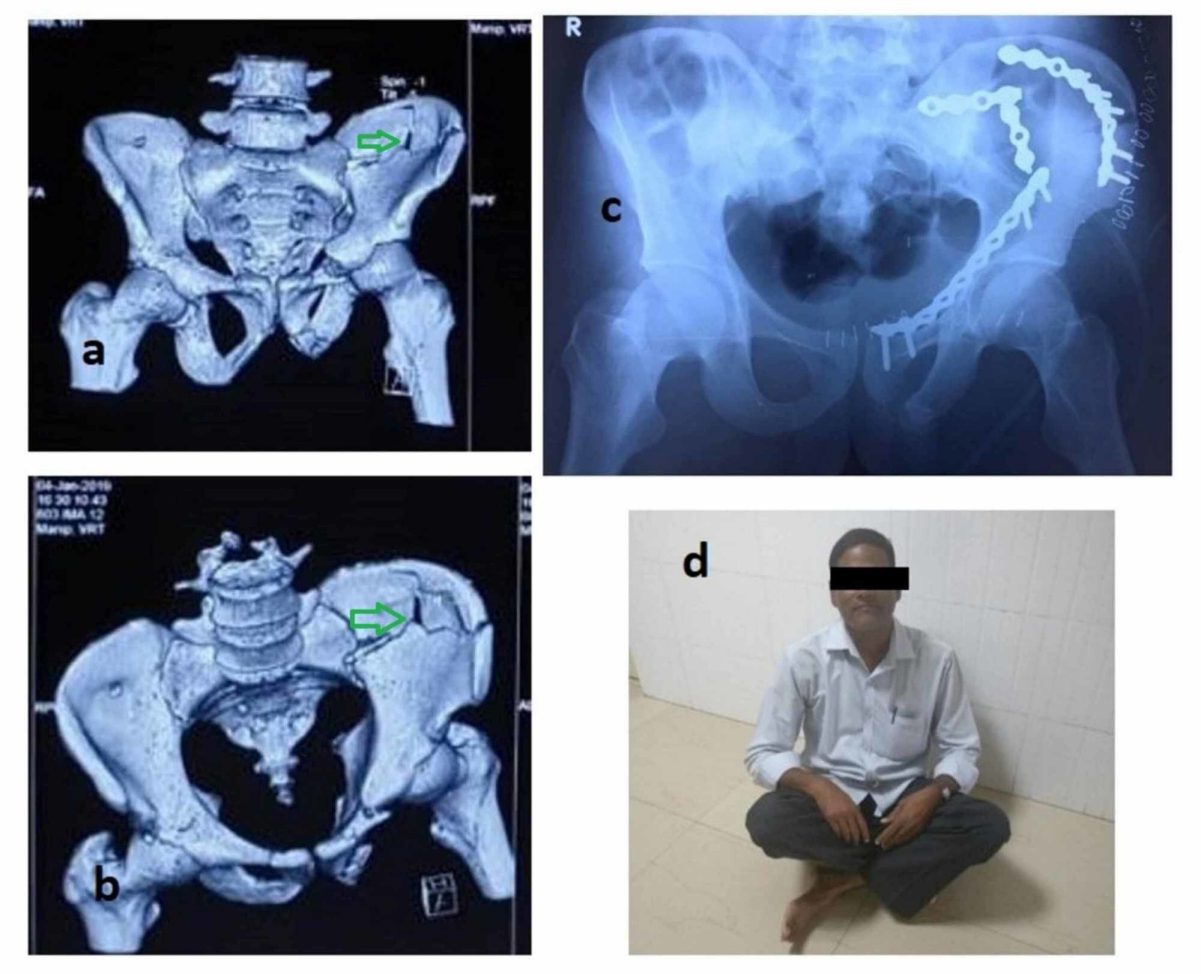
A case of anterior column fracture extending high in iliac blade (green arrows) with final postoperative X-rays and functional outcome a, b: preoperative CT of the pelvis with both hips; c: follow-up radiograph; d: follow-up clinical photograph of the patient sitting cross-legged CT: computed tomography

The association between postoperative reduction and HHS and NMAPS was not significant (p: 0.585 for HHS, p: 0.23 for NMAPS) when evaluated using the chi-squared test (Fisher's exact test). Similarly, the correlation by the Kendall correlation test was also not significant (tau = 0.14 for HHS and tau = 0.15 for NMAPS; p: >.0.05 for both). HHS and NMAPS were significantly correlated (tau = 0.43, p: <0.05). Some of the cases that were operated at our hospital are illustrated in Figures 1-4.

The intraoperative complication in the form of severe blood loss (>1,000 ml) was seen in one patient. There were no cases of vessel injury. Two patients developed superficial infections that improved conservatively. Three patients had iatrogenic obturator nerve palsy and recovered in three to four months. One of our initial patients had a direct inguinal hernia that required surgery, and two patients developed grade 2 arthritic changes during the follow-up period. One patient developed grade 3 bedsores that required skin grafting.

## Discussion

Acetabulum fractures are on the rise in developing countries, unlike in the western world where they have reached a plateau [12-13]. These fractures require absolute reduction to attain the best results as the hip joint is the major weight-bearing joint of the body [1-2]. Letournel et al. proposed that surgical management had a better outcome than the conservative approach [1]. However, the major challenge in developing countries, including India, is the lack of appropriately trained acetabular surgeons to address these complex injuries [12,14]. Less experienced surgeons often try to manage these fractures conservatively or refer them to other surgeons. Precious time is lost before the initial surgeon referral and often patients also procrastinate in making the decision [15]. Ultimately, a delayed presentation affects the functional outcome. Fracture patterns and displacements govern the surgical approach. Classically, the IL approach has been used as the anterior approach, and the results have been satisfactory [4-5]. Unfortunately, the learning curve for the surgeon is steep; reductions remain indirect, and complete visibility of all the three windows is difficult [16]. The patients are subjected to increased morbidity and complications, particularly in a beginner's hands. The intrapelvic Stoppa approach has the advantage of being safer in the learner's hands, providing direct visualization of bony structures, particularly the quadrilateral plate and posterior column that can be instrumented [17]. A lateral window, as described by Sagi et al., allows another space for instrumentation and is more useful for high anterior iliac blade fractures as well [9].

The mean age of our patients with acetabular fracture was 33.5 years, which contrasted with that of patients in the US and China, where the mean age of the patients was more than 40 years [3]. This was most probably due to the age pyramid and sedentary lifestyle of older patients in our country. The mean ISS in our series was 18.5, with more than 50% suffering from polytrauma. Motor vehicle accidents causing high-energy trauma are mostly responsible for this major injury involving multiple systems. In our country, the younger generation violates traffic rules more by indulging in rash driving, over-speeding, and not wearing safety gear such as helmets and seat belts. Other associated life-threatening injuries take priority over acetabular fractures that remain neglected, and this adds to their delayed referral and presentation. Paksoy et al. found associated both-columns fracture as the most common presentation (54%), followed by transverse with the posterior column fracture (19%) in their series [18]. Similarly, we also found that both-column and transverse patterns in the majority of the cases. Head impaction fracture was seen in transverse fractures and is a poor prognostic factor for functional outcomes.

The average time interval between injury and surgery is more than compared with those in other studies. This delay is caused by the time taken to reach the tertiary center of specialization. There is a lack of expertise in managing acetabular fractures among many practicing surgeons. This is more so in the anterior approach (IL), which is supposed to be riskier in comparison to the KL approach. Delayed presentation is also responsible for more operative time. We also experienced extended time in a few of our earlier cases in comparison to the later ones as we became familiarized. These were comparable with other studies, which signifies a smaller learning curve for this approach. One case, in particular, was an outlier, requiring more time, and there was significantly more blood loss than the rest. A tributary of the external iliac vein had been lacerated and was managed by pelvic packing for some time.

The functional result is a summation of the surgeon's experience, fracture pattern, associated injury, and radiological reduction of the fractures besides the patient's characteristics (age, BMI, etc.) [19,20]. Radiological assessment is best done by an X-ray. CT scans are not routinely recommended postoperatively in view of their cost and radiation hazard though they have become more sensitive [21]. Anatomical reduction leading to the congruent joint has been found to correlate with functional outcomes [22]. However, the communication of the weight-bearing dome (>3 fragments) causes difficulty in anatomical reduction [23]. In our study, the association and correlation between radiological reduction and association were not significant. This may be due to heterogeneity in our cohort-variable fracture pattern, the variable time interval between injury and surgery, associated head impaction fracture, and chondral injury. Marsh et al. have pointed out that initial irreversible chondral injuries are responsible for progression to arthritis [24]. One patient with an associated both-column fracture that was neglected had good functional outcomes because secondary congruity was achieved. We measured the functional outcomes using both HHS and modified NMAPS, in contrast to other studies, for higher inter-evaluator reliability [11]. When patients were grouped as acceptable and non-acceptable, we had a functional outcome of 74% in the HHS and 79% in NMAPS groups [12].

Our results are very close to those of the earlier published series of experienced surgeons. Hirvensalo et al. achieved functional results of HHS of >75 points in 80% of the patients [6]. Kim et al. attained good-to-excellent clinical results in 16 of 20 cases (80%) [22]. This clearly shows that the technique is easier and replicable even by beginner surgeons like us. Recently Guo et al. reported 100% acceptable (excellent and good) radiological and functional scores using Majeed scoring [16]. There is a paradigm shift towards the Stoppa approach in recent times, as highlighted by the number of annual publications. A meta-analysis by different authors comparing the IL to MSA has clearly shown better reduction, lower blood loss, and fewer complications in the MSA group even though the functional outcome is comparable [25-27].

Direct inguinal hernia was seen in our earlier cases. A large incision and inappropriate closure could be the reasons. Obturator nerve palsy occurred in three patients. This is because of neuropraxia in retraction, which recovered over time. Interestingly, in one study, Kim et al. found that obturator nerve palsy occurs in initial trauma with the displacement of the quadrilateral plate (>24 mm) [23]. One patient had associated spinal injury (ASAI B) and developed bedsores that required skin grafting. Paksoy et al. in their study on the Stoppa approach have concluded that the approach is imperative but is not without complications [18]. There were no cases of heterotopic ossification, and thromboembolic issues were seen in our series in contrast to other studies. Meena et al. (2016) and Yao et al. (2020) have reported that MSA has lesser complications than the IL approach [25,26]. Postoperative arthritis (PTA) has been reported variably by several authors [1,28]. Letournel et al. have reported that half of the patients develop PTA with anatomical reduction till up to 25 years, while those with imperfect reduction develop it to the extent of 80% by 10 years [1]. Hence, a close follow-up becomes mandatory.

The main limitation of our study is that it is a retrospective review of a small number of patients. Hence, we could not segregate the fracture patterns and perform a subgroup analysis to find their association with the final functional outcomes. The cohort was also heterogeneous regarding the time of presentation, a confounder for functional outcomes. Long-term follow-up for complications, particularly post-traumatic arthritis, is also required.

## Conclusions

The use of the MSA for the treatment of acetabular fractures leads to a good-to-excellent reduction in the majority of cases since it gives direct visualization of the quadrilateral plate and posterior column. The learning curve is smaller for less-experienced surgeons, resulting in fewer complications and better results. We recommend this technique as a viable alternative to the IL approach for anterior acetabular fixation.
